# Identification and Resolution of Drug-Related Problems among Childhood Cancer Patients in Ethiopia

**DOI:** 10.1155/2020/6785835

**Published:** 2020-03-16

**Authors:** Malede Berihun Yismaw, Haileyesus Adam, Ephrem Engidawork

**Affiliations:** ^1^Department of Pharmacology and Clinical Pharmacy, School of Pharmacy, College of Health Sciences, Addis Ababa University, P.O. Box 1176, Addis Ababa, Ethiopia; ^2^Department of Pediatrics, School of Medicine, College of Health Sciences, Addis Ababa University, P.O. Box 1176, Addis Ababa, Ethiopia

## Abstract

**Background:**

Even though medications play a major role in the cure, palliation, and inhibition of disease, they also expose patients to drug-related problems. Drug-related problems are frequent and may result in reduced quality of life, morbidity, and mortality.

**Objectives:**

The study was aimed to identify, characterize, and resolve drug-related problems in the Pediatric Hematology/Oncology ward of Tikur Anbessa Specialized Hospital, Addis Ababa, Ethiopia.

**Methods:**

A prospective observational study was conducted from 25 June to 25 October 2018 to assess DRPs on patients admitted at the pediatric hematology/oncology ward of Tikur Anbessa Specialized Hospital, which is the highest level governmental tertiary care hospital in Ethiopia. Data were obtained from patients' medical charts, physicians, patients/caregivers, pharmacists, and nurses. All the collected data were entered and analyzed using the Statistical Package for the Social Sciences version 25e. Descriptive statistics were used to represent the data.

**Results:**

Among the total 156 participants, DRPs were identified in 68.6% of the study subjects. Dosing problems which include dosage too low and high were the top ranking (39.3%) of all DRPs followed by needs additional therapy (27.2%) and nonadherence (14.0%). Systemic anti-infectives were the most common class of drugs involved in DRPs. Trimethoprim-sulfamethoxazole, methotrexate, vincristine, ondansetron, and metoclopramide were frequently involved in DRPs. The addition of drugs and change in drug dose were the two most proposed intervention types. Among the proposed interventions, 223 (92.15%) were fully accepted, 9 (3.72%) partially accepted, and 10 (4.13%) not accepted.

**Conclusion:**

DRPs are common among Pediatric Hematology/Oncology ward patients. The hospital should develop a pediatric dosing chart for the commonly prescribed medications to prevent drug-related morbidity and mortality. The integration of clinical pharmacists can mitigate risks associated with DRPs.

## 1. Introduction

The healthcare system of Ethiopia is structured into a three-tier system: primary, secondary, and tertiary levels of health care. The sector has shown remarkable progress involving the number of health, nutrition, and population indicators over the last decade [1]. Of the health programs that are given emphasis, clinical pharmacy services are among priority policy directions. The core clinical pharmacy activities delivered in the inpatient departments include undertaking medication reconciliation, making ward rounds, conducting morning sessions, and provision of discharge medication counseling in patients having varied clinical diagnoses including cancer [2].

Cancer is the second leading cause of death worldwide [3] with an estimated prevalence of 22.2 million people by the year 2030 [4]. The majority of this magnitude of cancer falls in developing countries [5]. In Ethiopia, national data on the prevalence and incidence of cancer are lacking. However, extrapolation from the Radiotherapy Centre of the Tikur Anbessa Specialized Hospital (TASH) estimates that there are 120,500 new cancer diagnoses in all age groups and about 6000 new childhood cancer diagnoses each year. Most of the pediatric cancer patients came with advanced stage and there is a high rate of treatment discontinuation that ultimately leads to high mortality rates [6].

Cancer pharmacotherapy is more complex and associated with many drug-related problems (DRPs) such as adverse effects, medication errors, interactions, and nonadherence [7]. Children are particularly susceptible to DRPs as there is a significant variation in organ development, weight, and body surface area (BSA), which can affect their ability to metabolize and excrete drugs effectively [8]. DRPs have been defined as “an event or circumstance involving drug therapy that actually or potentially interferes with desired health outcomes” [9].

According to Cipolle et al. [10] classification system, all patient problems involving medications can be categorized into one of the seven types of DRPs. It includes unnecessary drug therapy, need for additional drug therapy, ineffective drug, dosage too low, adverse drug reaction, dosage too high, and nonadherence.

DRPs are associated with many deleterious consequences. Some of these include emergency department visits, long term hospitalization, additional office visits, and long term care admissions. In addition to these, substantial costs are also allocated to resolve DRPs. For example, $177.4 billion annual expense was allocated for drug-related morbidity and mortality in the USA [11]. Likewise, a study from Australia reported that 4.3% of pediatric admissions were related to DRPs. Direct costs associated with DRPs have been reported to be £100,707 [12].

Prevention is the key to all aspects of health care. By assessing individual patient risks, it is also possible to prevent the occurrence of DRPs before their existence. However, it is not always possible to prevent the occurrence of DRPs. As a result, the evaluation of pharmacotherapy after its initiation is vital to detect DRPs and optimize treatment outcomes [13]. Different studies done elsewhere reported that clinical pharmacists' intervention may help to avoid DRPs and improve patients' therapeutic outcomes and quality of care [8, 12, 14–16]. Therefore, the objective of this study was to identify clinically significant DRPs and make an appropriate intervention in the pediatric hematology/oncology ward of TASH, Addis Ababa, Ethiopia.

## 2. Materials and Methods

### 2.1. Patient Selection

The study included all newly admitted patients to this ward and fulfilling the inclusion criteria during the study period. Refusal to participate, unconfirmed diagnosis, repeated admission, and waiting only for surgical management were the exclusion criteria. Ethical approval was obtained from the School of Pharmacy, College of Health Sciences, Addis Ababa University Ethics Review Board and permission letter was also obtained from the pediatric department. Informed consent from a care giver and assent from participants aged 12 years and above were also obtained. Confidentiality of the information of the study participants was ensured through anonymity and restricting data access.

### 2.2. Study Design and Setting

A prospective observational study was conducted for four months period from 25 June to 25 October 2018 to assess DRPs in the pediatric hematology/oncology ward of TASH. TASH is the only highest level referral center for critical and complicated health problems in the country. It is also the only cancer center for the entire country. It offers comprehensive health care services for around half a million patients per year through specialty clinics and inpatient service departments. It has over 700 beds and about 1700 professional and support staff in inpatient, outpatient, and emergency units. On average, the pediatric hematology/oncology ward gives services for around 260 patients per year.

### 2.3. Data Collection Techniques

The data abstraction format includes all pertinent information that is needed to deliver pharmaceutical care. The hospital has only paper-based patient records. Supplementary information and clarifications on some patient's medical information were obtained through discussion with the care giver and the physician. Adherence and administration related problems were assessed through observation and discussion with physicians, patients/care givers and nurses. In addition, the availability, strength, dosage form selection, and counseling issues of drugs were discussed with pharmacists. The patients were followed up on a daily basis.

Data were collected by trained data collectors (two pharmacists and one nurse). Suitability of the data abstraction format was assessed through in-depth discussion with members of the research team. The pretest was also done on 10 patients who were admitted to the pediatric hematology/oncology ward of TASH before data collection to ensure consistency of data collection format and appropriate modifications were made accordingly. Data were reviewed on a daily basis for accuracy and consistency.

Once the data were collected, appropriateness of medical therapy was evaluated using various references, including Medscape, Up-to-date 21.6 version, Micromedex, standard and updated text books, and specific guidelines from National Comprehensive Cancer Network (NCCN) and American Academy of Pediatrics (AAP) based on the updated daily patient and clinical characteristics. If there exists a discrepancy among the resources, the research team decides after searching other recently published article reports. Equations like modified Schwartz equation for creatinine clearance calculation, Du Bois method for BSA calculation, and Calvert formula for carboplatin dose calculation were used. The doses of cytotoxic medications were evaluated based on the Hematology/Oncology Pharmacy Association (HOPA) guideline [17].

The research team includes one pediatric oncologist, one pharmacologist, three clinical pharmacists, and one nurse. The research team was responsible for proposing possible intervention measures and subsequently communicated to either the oncologists/hematologists/residents/nurses/pharmacists or the patients/care givers by the two clinical pharmacists (data collectors). The identified DRPs were recorded and classified using the DRP registration format of Cipolle et al. [10] and the status of interventions was documented. In addition, drugs associated with DRPs were classified using the Anatomical Therapeutic Chemical (ATC) classification system [18].

### 2.4. Statistical Analysis

The collected data were categorized, coded, entered, and analyzed using the Statistical Package for the Social Sciences (SPSS) version 25 software. Descriptive statistics such as mean, median, interquartile range (IQR), cross-tabulation, and frequencies were used to present the data.

## 3. Results

### 3.1. Sociodemographic and Clinical Data

Of the 176 patients admitted during the study period, data for 20 patients were excluded in the final analysis (16 patients were waiting for only surgery, one patient discharged against medical advice, and three were readmitted cases). The sociodemographic and clinical characteristics of the study population are described in Table 1. The majority (62.8%) of them were males, 87.2% were children aged from 1 to 10 years with a mean age of 4.2 years, and comorbid medical conditions were present in 16.0% of the study participants. The most common comorbid condition diagnosed was hypertension.

The median hospital stay of the participants was 9 (IQR = 6–19) days and the total number of patient days was 2203. A total of 1887 drug prescriptions were prescribed for 156 patients and the median number of drugs prescribed in the study population was 11 (IQR = 8–15).

### 3.2. Type of Cancer Diagnosis

**Figure 1:**
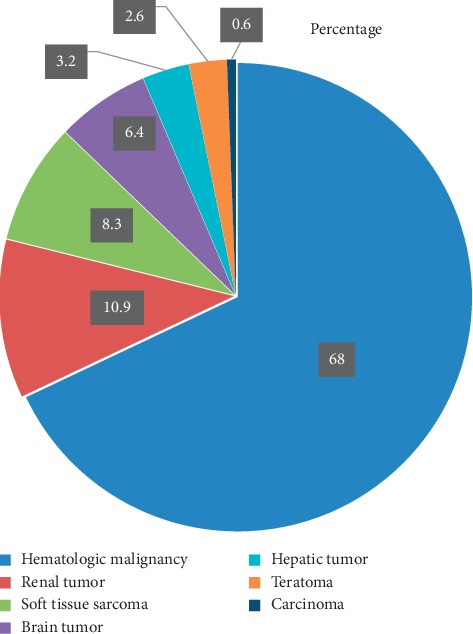
Types of cancer diagnosed at the pediatric hematology/oncology ward of Tikur Anbessa Specialized Hospital, Addis Ababa, Ethiopia, 25 June- 25 October 2018.

Hematologic malignancies were the most common (68%) types of cancer diagnosed (Figure 1). A renal tumor (10.9%) was the second most, and carcinoma (0.6%) was the least commonly diagnosed cancer. The specific hematologic malignancies include Non-Hodgkin lymphoma (29.5%), Acute lymphoblastic leukemia (20.5%), Acute myeloblastic leukemia (14.7%), and Hodgkin lymphoma (3.2%), whereas Squamous cell cancer of the tongue was the only carcinoma diagnosed.

### 3.3. Prevalence and Types of Drug-Related Problems

A total of 257 DRPs were identified from 107 (68.6%) of the study participants, out of which 1 DRP was found in 40 (25.6%), 2 DRPs in 31 (19.9%), and 3 or more DRPs in 36 (23.1%) of patients. Dosing problems, which included dosage too low and high, were the top ranking (39.3%) types of DRPs identified in the study subjects followed by the need for additional drug therapy. Prescribing ineffective doses of drugs was the most common (47.5%) cause of dosing problem, whereas the need for prophylaxis therapy to reduce the risk of developing new disease conditions was the common (70%) cause of the need for additional therapy. However, DRPs related to adverse drug reaction (ADR) and ineffective drugs accounted for less than 10%. The type and number of DRPs identified were depicted in Table 2.

### 3.4. Drugs and Drug Classes Involved in Drug-Related Problems

**Figure 2:**
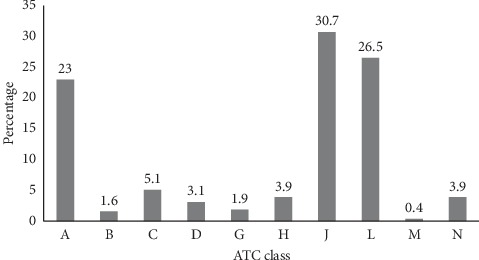
Percentage of drug-related problems according to the Anatomical Therapeutic Chemical classification of the drug. A: Drugs acting on alimentary tract and metabolism, B: Drugs acting on blood and blood forming organs, C: Drugs acting on cardiovascular system, D: Dermatologic drugs, G: Drugs acting on genitourinary system and sex hormones, H: Systemic hormonal preparations, excluding sex hormones and insulin, J: Anti-infectives for systemic use, L: Antineoplastic and immunomodulating agents, M: Drugs acting on musculoskeletal system, and N: Drugs acting on nervous system.

Anti-infectives for systemic use (ATC group J) were the most common (30.7%) drug class involved in DRPs followed by antineoplastic and immunomodulating agents (ATC group L, 26.5%) and drugs acting on alimentary tract and metabolism (ATC group A, 23.0%) (Figure 2).

A total of 57 drugs were involved in different types of DRPs. Among these, the most frequently involved drugs were Trimethoprim-sulfamethoxazole (TMP/SMX) (35), methotrexate (25), vincristine (12), ondansetron (12), and metoclopramide (11) (Table 3). Needs additional drug therapy with TMP/SMX and nonadherence of methotrexate were the more frequent identified DRPs, which accounted for 16.3% of all DRPs.

### 3.5. Interventions for Drug-Related Problems

**Figure 3:**
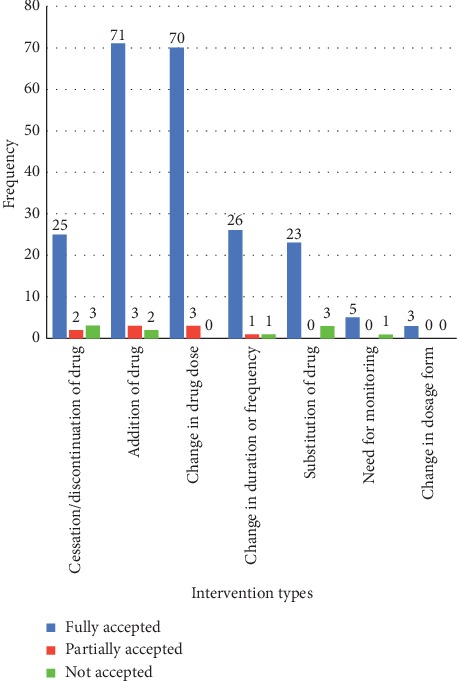
Type of intervention given and its outcome. Partially accepted: the recommended intervention is accepted and implemented with modification or not implemented.

Appropriate interventions were made to correct the identified DRPs. Of the 257 DRPs, the intervention was made for 242 (94.2%) of the identified DRPs. The addition of drugs (76,31.4%) and change in drug dose (73, 30.2%) were the two most frequently provided intervention types as shown in Figure 3. The rest of the interventions were cessation/discontinuation of the drug, change in duration or frequency, the substitution of the drug, the need for monitoring, and change in the dosage form. Among the provided interventions, 223 (92.15%) were fully accepted, while 9 (3.72%) partially accepted and 10 (4.13%) not accepted.

## 4. Discussion

The goal of pharmacotherapy is to attain definite therapeutic outcomes, minimize medication risks, and improve patients' quality of life. Inappropriate use of medications is common around the globe and may expose patients to DRPs [13, 19]. Different studies have shown that clinical pharmacists can effectively prevent and resolve these DRPs [8, 14–16, 20, 21]. Therefore, this study was carried out to identify DRPs and make an appropriate intervention in pediatric hematology/oncology ward patients.

In the present study, 257 DRPs were identified among 107 (68.6%) of the included patients, giving an overall frequency of 1.65 DRPs per patient or an average of 2.4 DRPs in those patients with DRPs. Although data in the literature are scarce in the area, comparison with available data indicates that the frequency of DRPs was higher than a study done in a similar setting, where 0.6 DRPs per patient was reported [22]. The prevalence was also higher compared to other studies conducted in the pediatric wards of local (32%) and overseas (21%) hospitals [23, 24]. Studies performed in adult cancer patients also reported either a lower (55%) [25] or higher (93.8%) [26] prevalence than the current study. The difference could be attributed to differences in training levels of prescribers, availability of support systems, and composition of the health care team in these hospitals. Nonetheless, similar prevalence rates (66–75%) were also reported in different studies [20, 27–30].

The most frequently encountered DRPs were inappropriate dosing (high and low dose) followed by needs additional drug therapy and nonadherence to the prescribed medications. In line with this, several studies have also reported dosing problems (high and low dose) to be the most frequently (34.9%–61.8%) encountered DRPs in their settings [24, 31, 32]. The drugs more associated with dosing problems in this study included TMP/SMT, vancomycin, vincristine, metoclopramide, cimetidine, furosemide, and doxorubicin. For example, a 4-year-old female patient weighing 18 kg diagnosed with acute lymphocytic leukemia (ALL) was taking trimethoprim/sulfamethoxazole (TMP-SMX) 80 mg po 3x/week for pneumocystis carinii pneumonia (PCP) prophylaxis and intervention was made to increase to 480 mg po/week as 5 mg/kg of TMP part OR 30 mg/kg of the combination drug is recommended. Another female patient weighing 10 kg diagnosed with Wilms tumor and neutropenic fever (NF) was taking meropenem 200 mg iv TID and Vancomycin 50 mg iv TID as part of the NF regimen. Her Creatinine Clearance was calculated to be 68.8 ml/min and vancomycin dose was increased to 150 mg iv TID as per the recommendation. In the first case, the prescriber used 5 mg/kg for the combination drug and 15 mg/kg of vancomycin for daily dose instead of using 15 mg/kg/dose or 40–60 mg/kg/day for the second case.

Dosing problems result in reduced efficacy or safety problems which leads patients to drug-related morbidity and mortality. The weight-based dosing calculations, fractional dosing, and the need for decimal and incorrect recording of patients' weights result in inappropriate dosing in pediatrics as compared to adult population [33]. In addition, inadequate knowledge of prescribers and the absence of a pediatric drug dosing chart in the setup resulted in the occurrence of dosing errors. Therefore, the high prevalence of dosing problems in the present study would make this an important area requiring further attention. Needs additional drug therapy was also common DRP type, which is concordant with a study done at the Australian pediatric teaching hospital [34].

ADR and ineffective drugs were the least prevalent DRP types, which accounted for 5.4% and 4.3%, respectively. In contrast, ADR was identified as the most frequently encountered DRP in other studies both in pediatric as well as adult cancer patients [29, 35]. ADRs are strongly connected to cancer chemotherapy. Since the majority of chemotherapeutic drugs cannot differentiate between cancer and normal cells, many ADRs are unavoidable and usually accepted by health care providers and patients [7]. The lower ADR prevalence in the present study might be due to the nonreporting of ADRs that were managed appropriately.

In contrast to our finding, treatment effectiveness was also the major (50.2%) type of DRP, which was followed by treatment safety (24.7%) in a study conducted in Northern Cyprus [25]. As the country has limited pharmaceutical products in circulation, the study evaluated the treatment based on the available drugs in the national drug list, which could probably justify the lower prevalence of ineffective drug use.

Methotrexate was used as a backbone and also a central nervous system (CNS) prophylactic agent of choice in many of our protocols but stock out of this medication was seen repeatedly in our setup and accounted for half of the DRPs related to methotrexate. Methotrexate was the second frequent (9.7%) of all drugs associated with DRPs. Essential drugs shortage including chemotherapeutic agents is more common in low-middle income countries. Unavailability of these drugs is majorly seen in Pediatric oncology wards [36].

In order to identify the most common drug classes associated with DRPs, the ATC classification system was used [18]. Based on this classification system, anti-infectives for systemic use (ATC group J) was the most common (30.7%) drug class associated with DRPs in this study. This finding is in agreement with other studies done in patients with hematology malignancies [37] as well as other patients [22, 24, 30, 31, 38]. Anti-infective drugs encompass the largest number of drug classes widely prescribed in TASH and associated with DRPs needing pharmaceutical interventions. The second common drug class associated with DRPs was antineoplastic and immunomodulating agents (ATC group L, 26.5%), which appeared to be much higher than a study done in a similar setting in France (3.9%) [37]. The most identified problem in this study associated with antineoplastic and immunomodulating agents was BSA calculation error, which could possibly be attributed to differences in training levels of residents and the absence of clinical pharmacists.

Intervention was provided for 242 (94.2%) of all DRPs identified. Intervention was not made for the rest due to different reasons, such as unavailability of the proposed/substitute drug, discharge, or death of the patient before the recommendation was made. The addition of a drug, which accounted for about 31.4%, was the most common recommendation made, and this is consistent with an Indian study (29.3%) [27]. The second most common intervention recommended was dose adjustment (30.2%), and a similar rate was also reported in a Cote d'Ivoire study (32%) [31]. Interventions were fully accepted in 223 (92.15%) and not accepted in 10 (4.13%) of the cases. Only 3 of 30 measures proposed for cessation/discontinuation of the drug were not accepted. Among DRPs associated with the addition of drug 2 of 76 measuers were not accepted, while 74 of measures were either partially or fully accepted. Only 1 of 28 recommendations given to change in duration, 3 of 26 measures to substitute a drug, and 1 of the 6 measures to do monitoring parameters were not accepted. The reasons for nonacceptance includes fearing of the legal issues, absence of local guideline supporting the recommendations, and not trusting proposed evidence.

Various acceptance rates have been reported from different countries. The current rate is similar with the National Cancer Centre of Singapore (93%) [39], slightly lower than that of the Cote d'Ivoire (94.8%) [31] and France (96%) [37], and higher than that of Norway (75%) [20], India (86.6%) [40], and South Korea (88.3%) [41]. However, a very low acceptance rate (37.4%) was also reported in a study conducted in Iraqi hospitals [42]. The difference could be attributed to differences in hospital settings and training levels of clinical pharmacists to give evidence-based recommendations and existing composition of the health care team in these hospitals. The high acceptance rate in our setup might also be due to the existence of a critical evaluation of each recommendation by the research team before intervening. In general, clinical pharmacists' acceptance rate was high (92.15%) in our setup, which indicates the great recognition and acceptance of clinical pharmacists in the inpatient setup.

### 4.1. Study Limitations

Firstly, the study did not show the overall incidence of DRPs for a patient in his/her hospital stay (admission to discharge) rather, it shows DRPs within the study period. Secondly, the intervention given for the identified DRPs may, to some extent, affect the incidence of DRPs in the subsequent study subjects. Thirdly, the hospital is very specialized and the result may not be generalized to all hospitals.

## 5. Conclusion

DRPs are common among pediatric hematology/oncology ward patients. Dosing problems are more frequent than other types of DRPs. The hospital should develop a pediatric dosing chart for the commonly prescribed medications to prevent drug-related morbidity and mortality. Integration of clinical pharmacists can effectively prevent, identify, and resolve clinically significant DRPs. In general, to decrease DRPs and improve the quality of health care, the hospital requires a coordinated intervention from all concerned bodies and need to assign clinical pharmacists in wards.

## Figures and Tables

**Table 1:** Sociodemographic and clinical characteristics of patients at the pediatric hematology/oncology ward of Tikur Anbessa Specialized Hospital, Addis Ababa, Ethiopia, 25 June to 25 October 2018.

Variable	Category	Number (%)
Age	≤1 year	10 (6.4)
	>1 year to ≤5 years	71 (45.5)
	>5 years to ≤10 years	65 (41.7)
	>10 years to ≤15 years	10 (6.4)

Sex	Male	98 (62.8)
	Female	58 (37.2)

Residence	Urban	86 (55.1)
	Rural	70 (44.9)

Family history of cancer	Yes	6 (3.8)
	Not known	150 (96.2)

Caregiver education	No formal education	21 (13.5)
	Grade 1–8	55 (35.2)
	Grade 9–12	42 (26.9)
	College and above	38 (24.4)

Hospital stay	≤10 days^*∗*^	91 (58.3)
	>10 days	65 (41.7)

Comorbid conditions	Yes	25 (16)
	Hypertension	20 (12.8)
	Retroviral infection	4 (2.6)
	Congestive heart failure	1 (0.6)
	No	131 (84.0)

Neutropenic fever presence	Yes	49 (31.4)
	No	107 (68.6)

Total number of prescriptions per patient	≤10 drug prescriptions	58 (37.2)
	>10 to ≤20 drug prescriptions	84 (53.8)
	>20 drug prescriptions	14 (9.0)

^*∗*^Short hospital stay is defined as a hospital stay of less than or equal to 10 days in the ward.

**Table 2:** Types of drug-related problems identified at the pediatric hematology/oncology ward of Tikur Anbessa Specialized Hospital, Addis Ababa, Ethiopia, 25 June–25 October, 2018.

Types of DRPs	Causes of DRPs	No. of DRPs	Total	(%)
Unnecessary drug therapy	Duplicate therapy	12	25	9.7
	No medical indication at this time	13		

Needs additional therapy	Preventive therapy	49	70	27.2
	Untreated condition	20		
	Synergistic therapy	1		

Ineffective drug	More effective drug available	6	11	4.3
	Dosage form inappropriate	5		

Dosage too low	Ineffective dose	48	60	23.3
	Frequency inappropriate	10		
	Duration inappropriate	2		

Adverse drug reaction	Undesirable effect	6	14	5.5
	Drug interaction	1		
	Incorrect administration	1		
	Dosage increase/decrease too fast	6		

Dosage too high	Dose too high	28	41	16.0
	Needs additional monitoring	3		
	Frequency too short	7		
	Duration too long	3		

Nonadherence	Does not understand instructions	6	36	14.0
	Cannot afford drug product	1		
	Patient prefers not to take	5		
	Patient forgets to take	1		
	Drug product not available	22		
	Cannot swallow/administer drug	1		

**Table 3:** Top ten specific drugs associated with drug-related problems at the pediatric hematology/oncology ward of Tikur Anbessa Specialized Hospital, Addis Ababa, Ethiopia, 25 June- 25 October, 2018

Drug name	Drug-related problem category							Total
	Unnecessary drug therapy	Needs additional therapy	Ineffective drug	Dosage too low	Adverse drug reaction	Dosage too high	Nonadherence	
TMP/SMX	1	20	1	11	0	0	2	35
Methotrexate	0	2	0	0	0	1	22	25
Vincristine	3	1	0	6	0	2	0	12
Ondansetron	2	7	0	0	1	2	0	12
Metoclopramide	0	5	1	4	0	1	0	11
Doxorubicin	1	3	1	2	0	3	0	10
Cimetidine	1	0	2	4	0	2	0	9
Ceftriaxone	4	0	1	1	0	1	0	7
Diphenhydramine	1	2	0	2	0	2	0	7
KCl	0	4	0	0	0	1	1	6

KCl: potassium chloride, TMP/SMX: trimethoprim-sulfamethoxazole.

## Data Availability

The dataset used to support the finding of this study will be available upon request.

## Supplementary Materials

Supplementary Materials The data abstraction format includes the sociodemographic and clinical characteristics of each participant that are used to deliver pharmaceutical care. In addition, drug-related problem (DRP) registration format of Cipolle et al. was used. The Cipolle et al. DRP classification system includes the seven types of DRPs (unnecessary drug therapy, need for additional drug therapy, ineffective drug, dosage too low, adverse drug reaction, dosage too high, and nonadherence) with their respective causes. Drugs associated with DRPs are also classified using the WHO-Anatomical Therapeutic Chemical (ATC) Classification system. In the ATC classification system, drugs are divided into different groups according to the organ or system on which they act and their therapeutic, pharmacological, and chemical properties. The data abstraction tool also includes the types of interventions given and its outcome for each DRP.
