# Supplementary material for: Identification and Resolution of Drug-Related Problems among Childhood Cancer Patients in Ethiopia
Source: J Oncol. 2020 Mar 16;2020:6785835. doi: 10.1155/2020/6785835 (PMC7102482; doi:10.1155/2020/6785835)
Supplement: Supplementary Materials — The data abstraction format includes the sociodemographic and clinical characteristics of each participant that are used to deliver pharmaceutical care. In addition, drug-related problem (DRP) registration format of Cipolle et al. was used. The Cipolle et al. DRP classification system includes the seven types of DRPs (unnecessary drug therapy, need for additional drug therapy, ineffective drug, dosage too low, adverse drug reaction, dosage too high, and nonadherence) with their respective causes. Drugs associated with DRPs are also classified using the WHO-Anatomical Therapeutic Chemical (ATC) Classification system. In the ATC classification system, drugs are divided into different groups according to the organ or system on which they act and their therapeutic, pharmacological, and chemical properties. The data abstraction tool also includes the types of interventions given and its outcome for each DRP. [file 6785835.f1.docx]

# Annex I

**Data abstraction format**

1. **Socio demographic information**

Code number _______________ Sex: _____Wt.: ______Height: ______BSA: ______

Current age___________________ Age at diagnosis_______________________

Date of admission: __________________________________

Resident: urban rural

Family history of cancer yes no

Caregiver education No formal education Grade 1-8

Grade 9-12 College and above

1. Clinical characteristics

Working diagnosis

| Date |  |  |  |  |  |
| --- | --- | --- | --- | --- | --- |
| Diagnosis |  |  |  |  |  |

Cancer diagnosis_________________________________________

Number of total comorbidities__________________

Follow up start date ______________________ Follow up stop date ________________ Hospital stay: ______________________________

Past Medications: _______________________________________________________________ ______________________________________________________________________________

Immunization History: ___________________________________________________________ ____________________________________________________________________________________________________________________________________________________________

Review of Systems (ROS) (abnormal findings only):

__________________________________________________________________________________________________________________________________________________________________________________________________________________________________________________________________________________________________________________________________________________________________________________________________________________________________________________________________________________________________________________________________________________________________

Abnormal organ function tests & electrolytes by date

________________________________________________________________________________________________________________________________________________________________________________________________________________________________________________________________________________________________________________________________________________________________________________________________________________________________________________________________________________________________________________________________________________________________________________________________________________________________________________

Pertinent investigations

______________________________________________________________________________________________________________________________________________________________________________________________________________________________________________________________________________________________________________________________________________________________________________________________________

CBC with differentials

| Lab. Parameters | Normal  Range | Date of test | | | | | |
| --- | --- | --- | --- | --- | --- | --- | --- |
|  |  |  |  |  |  |  |  |
| WBC |  |  |  |  |  |  |  |
| Lymphocytes |  |  |  |  |  |  |  |
| Neutrophils |  |  |  |  |  |  |  |
| PLT count |  |  |  |  |  |  |  |
| RBC |  |  |  |  |  |  |  |
| Hgb |  |  |  |  |  |  |  |
| Hct |  |  |  |  |  |  |  |
| MCV |  |  |  |  |  |  |  |
| MCHC |  |  |  |  |  |  |  |
| ANC |  |  |  |  |  |  |  |

Vital sign (abnormal values, start from recent results)

**____________________________________________________________________________________________________________________________________________________________________________________________________________________________________________________________________________________________________________________________________________________________________________________________________________________________________________________________________________________________________________________________________________________________________________________________________________________________________________________________________________________________________________________________________________________________________________________________________________**

Medication Registration Form

| Date | Disease  condition | Medication/s given | Dosage regimen | Length  of  therapy | Comment  (DRP) |
| --- | --- | --- | --- | --- | --- |
|  |  |  |  |  |  |
|  |  |  |  |  |  |
|  |  |  |  |  |  |
|  |  |  |  |  |  |
|  |  |  |  |  |  |
|  |  |  |  |  |  |
|  |  |  |  |  |  |
|  |  |  |  |  |  |
|  |  |  |  |  |  |
|  |  |  |  |  |  |
|  |  |  |  |  |  |
|  |  |  |  |  |  |
|  |  |  |  |  |  |

Total number of drugs prescribed during study period ___________________

**Is there drug related problem**

- **Yes**
- **No**

1. **Drug related problem registration format of Cipole et al.**

| **1.Unnecessary drug therapy**   - Duplicate therapy - No medical indication at thistime - Nondrug therapy more appropriate - Addiction/recreational drug use - Treating avoidable adverse reaction |
| --- |
| **2. Needs additional therapy**   - Preventive therapy - Untreated condition - Synergistic therapy |
| **3. Ineffective drug**   - More effective drug available - Condition refractory to drug - Dosage form inappropriate - Drug not indicated for condition |
| **4. Dosage too low**   - Ineffective dose - Needs additional monitoring - Frequency inappropriate - Drug interaction - Incorrect storage - Duration inappropriate |
| **5. Adverse drug reaction**   - Undesirable effect - Drug interaction - Incorrect administration - Allergic reaction - Dosage increase/decrease too fast |
| **6. Dosage too high**   - Dose too high - Needs additional monitoring - Frequency too short - Duration too long - Drug interaction |
| **7. Non-adherence**   - Does not understand instructions - Cannot afford drug product - Patient prefers not to take - Patient forgets to take - Drug product not available - Cannot swallow/administer drug |

| Medication/s with DRP/s   1. ___________________________________________ 2. ___________________________________________ 3. ___________________________________________ 4. ___________________________________________ 5. ___________________________________________ 6. ___________________________________________ 7. ___________________________________________ 8. ___________________________________________ 9. ___________________________________________ 10. ___________________________________________ |
| --- |

**Medication/s class with DRP/s**

- A, Alimentary tract and metabolism
- B, Blood and blood forming organs
- C, Cardiovascular system
- D, Dermatologicals
- G, Genito-urinary system and sex hormones
- H, Systemic hormonal preparations, excluding sex hormones and insulins
- J, Antiinfectives for systemic use
- L, Antineoplastic and immunomodulating agents
- M, Musculo-skeletal system
- N, Nervous system
- P, Antiparasitic products, insecticides and repellents
- R, Respiratory system
- S, Sensory organs
- V, Various

1. **Interventions on DRP/s**

Type of intervention given

- Cessation of drug
- Addition of drug
- Change in drug dose
- Change in duration or frequency
- Substitution of drug
- Need for monitoring
- Change in dosage form
- No recommendation

Intervention outcome

- Fully accepted
- Partially accepted
- Not accepted
- Not applicable

Intervention respondent

- Physician
- Nurses
- Patients/caregiver
- Pharmacist
